# Central Venous Oxygen Saturation/Lactate Ratio and Prediction of Major Adverse Events After Pediatric Heart Surgery

**DOI:** 10.21470/1678-9741-2020-0521

**Published:** 2021

**Authors:** Victória Helena Stelzer Rocha, Paulo Henrique Manso, Fabio Carmona

**Affiliations:** 1 Department of Pediatrics, Faculdade de Medicina de Ribeirão Preto, Universidade de São Paulo, Ribeirão Preto, São Paulo, Brazil.

**Keywords:** Congenital Heart Disease, Cardiac Surgery, Pulmonary Gas Exchange, Sensitivity and Specificity, Heart Arrest, Incidence

## Abstract

**Introduction:**

Major adverse events (MAE) are unexpected but undesirably frequent after pediatric congenital heart surgery and contribute to poorer outcomes. The aim of this study was to test the predictive value of a ratio between central venous oxygen saturation and arterial lactate (ScvO2/lactate) for MAE after pediatric congenital heart surgery in a Brazilian university hospital.

**Methods:**

We conducted a retrospective observational study in a tertiary care university hospital, including 194 infants and children submitted to surgery for congenital heart disease. The predictive value of ScvO2, lactate, and ScvO2/lactate ratio were assessed by the area under the receiver operating characteristics curve (AUC), sensitivity, specificity, positive predictive value (PPV), and negative predictive value (NPV).

**Results:**

The incidence of MAE was 16% — cardiac arrest/death, unplanned reoperation, and low cardiac output syndrome were the most common events. Overall, ScvO2/lactate ratio discriminated patients with and without MAE very well (AUC 0.842), performing better than either variable alone, with sensitivity of 48%, specificity of 94%, PPV of 60%, and NPV of 91%.

**Conclusion:**

A ScvO2/lactate ratio > 5 can accurately identify patients at low risk of MAE after pediatric congenital heart surgery, with very good specificity and NPV, but poor sensitivity and PPV.

**Table:** Abbreviations, acronyms & symbols

AUC	= Area under the receiver operating characteristics curve
CI	= Confidence interval
CPB	= Cardiopulmonary bypass
cTnI	= Cardiac troponin I
IL	= Interleukin
LCOS	= Low cardiac output syndrome
MAE	= Major adverse events
NPV	= Negative predictive value
PPV	= Positive predictive value
RACHS-1	= Risk Adjustment for Congenital Heart Surgery 1
ROC	= Receiver operating characteristics
ScvO_2_	= Central venous oxygen saturation
SD	= Standard deviation
VIS	= Vasoactive-inotropic score

## INTRODUCTION

Major adverse events (MAE) are unexpected but undesirably frequent after pediatric congenital heart surgery and contribute to poorer outcomes. A MAE can be defined as cardiac arrest (with or without extracorporeal life support), emergency chest reopening or reoperation, and death^[1]^. Identifying patients at risk for MAE is challenging, but it could help physicians and nurses to monitor and allocate more resources to specific patients to prevent or rapidly address and treat a MAE. This has been attempted with the use of clinical examination (capillary refill time, pulses, urine output, core-toe temperature gradient), classical (heart rate, arterial blood pressure, central venous pressure, etc) and more advanced (cardiac index, systemic or pulmonary vascular resistance) hemodynamic variables, and laboratorial tests (lactate, base excess, arterial or central venous oxygen saturation [ScvO_2_]), but these approaches lacked sensitivity^[1,2]^.

Arterial lactate and ScvO_2_ are indirect markers of hypoxia, reflecting poor tissue perfusion, and exhibit higher sensitivity and specificity for predicting MAE than other variables do, especially after pediatric congenital heart surgery. However, when isolated, they still have low accuracy, but a combination of these two markers (a ratio between ScvO_2_ and arterial lactate, named ScvO_2_/lactate ratio), when < 5, showed a positive predictive value (PPV) of 93.8% for MAE, as well as 78.9% sensitivity and 90.5% specificity^[1]^. More recently, the vasoactive-inotropic score (VIS) was associated with poor outcomes after pediatric congenital heart surgery^[3]^.

However, the predictive value of ScvO_2_/lactate ratio for MAE after pediatric congenital heart surgery, with a cutoff value of 5, has not been validated in other populations. Therefore, we aimed to retrospectively assess the predictive value of ScvO_2_/lactate ratio for MAE after pediatric congenital heart surgery in a Brazilian university hospital. We hypothesized that a low ScvO_2_/lactate ratio would have a PPV of 90% or more for MAE.

## METHODS

This was a retrospective observational study carried out in a single center, a Brazilian tertiary care university hospital, the Hospital das Clínicas, Faculdade de Medicina de Ribeirão Preto, Universidade de São Paulo. The study followed the Brazilian regulations for research on human subjects^[4]^, it was approved by the local institutional review board (Research Ethics Committee HCFMRP-USP, protocol #CAAE: 99316918.0.0000.5440), and informed consent was waived. This study followed the STROBE recommendations for observational studies (https://www.strobe-statement.org/).

All patients < 18 years old submitted to congenital heart surgery between 2015 and 2017 were eligible for the study. The inclusion criteria were age < 18 years, gestational age ≥ 37 weeks, body weight ≥ 2 kg, and being submitted to open heart surgery. The exclusion criteria were death in the operation room, lack of information on medical records, and not having ScvO_2_ or lactate measured within 12 hours after surgery.

Data were retrieved from the electronical medical records, including demographic, clinical, surgical, and laboratorial data. An anonymized database was created using the Research Electronic Data Capture (or REDCap)^[5]^. Our primary outcome was the occurrence of a MAE within 48 hours after surgery — at least one of the following: cardiac arrest, unplanned reoperation, emergency chest reopening, low cardiac output syndrome (LCOS), or death. Unplanned reoperation was defined as the need for an additional, unanticipated surgical procedure or revision as a result of a significant postoperative residual lesion. Emergency chest reopening was defined as the need of sternum opening for exploration, bleeding control, or to alleviate pressure on the mediastinum. LCOS was clinically defined as the presence of altered mental status, mottled skin, cold extremities, prolonged capillary refill time (> 2 seconds), and weak pulses.

All ScvO_2_ measurements were done in venous blood drawn from catheters placed in the superior vena cava or in the right atrium (in the absence of left-to-right shunt). Lactate was measured in blood drawn from indwelling arterial lines already in place or from arterial puncture. The ScvO_2_/lactate ratios were calculated using paired measurements (within the same hour). The first values obtained within 0-6, 6-12, 12-24, and 24-48 hours after surgery were used for analysis, as well as the worst values within the first 48 hours (lowest for ScvO_2_ and ScvO_2_/lactate ratio, and highest for lactate). In all patients, only measurements made before the occurrence of MAE were considered in the analysis.

### Statistical Analysis

All results were described by means and standard deviations, medians and interquartile ranges, or counts (percentages). The continuous independent variables were categorized, and the best cutoff points for each one were determined by adjusting receiver operating characteristics (ROC) curves. The areas under the ROC curves (AUC), sensitivity, specificity, PPV, and negative predictive value (NPV) were also calculated. The individual contributions of each independent variable to the prediction of MAE were assessed by adjusting a multiple log-binomial regression model. The significance level was set at 0.05. Statistical packages JASP version 0.12.2 (JASP Team), Statistical Package for the Social Sciences (SPSS Inc. Released 2008, SPSS Statistics for Windows, Version 17, Chicago: SPSS Inc.), and Stata 14.0 (StataCorp, 2015, Stata Statistical Software: Release 14, College Station, TX: StataCorp LP.) were used.

## RESULTS

**Fig. 1:**
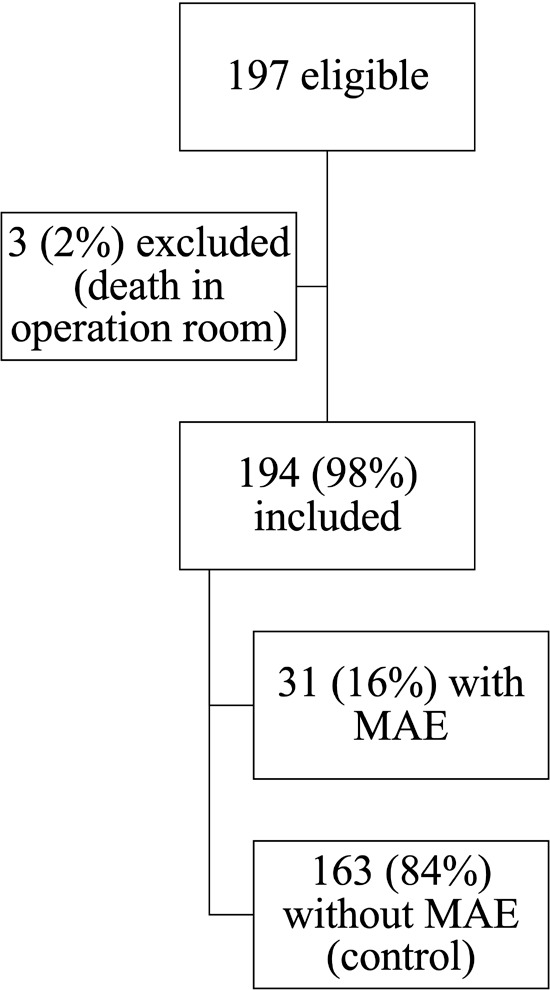
Flowchart of patient recruitment and inclusion. MAE=major adverse event.

A total of 197 patients were eligible for the study, but only 194 (98%) were included (Figure 1). The reason for exclusion of three patients was death at the operation room. The overall incidence of MAE was 16% (31 patients). The demographic, clinical, and surgical characteristics of all patients are depicted in Table 1. In short, patients who experienced a MAE were younger, more complex, and had higher VIS at the end of surgery and 12 hours after that. The types of MAE we observed are listed in Table 2.

**Table 1:** Demographic, clinical, and surgical characteristics of all included patients, according to the occurrence of a major adverse event (MAE) within 48 hours after surgery.

Characteristic	Control (n=163)	MAE (n=31)	*P*-value
Gender, male, n (%)	102 (63%)	16 (52%)	0.252
Age, months (mean ± SD)	34±52	14±43	0.044
RACHS-1 category, n (%)			0.031
1	49 (36%)	2 (13%)	
2	34 (25%)	2 (13%)	
3	44 (33%)	9 (56%)	
4	8 (6%)	3 (19%)	
VIS at end of surgery (mean ± SD)	11.4±15.4	21.1±24.1	0.004
VIS 12 hours after end of surgery (mean ± SD)	9.6±10.8	25.4±23.3	< 0.001
Cardiopulmonary bypass duration, minutes (mean ± SD)	118±55	136±76	0.160

RACHS-1=Risk Adjustment for Congenital Heart Surgery 1; SD=standard deviation; VIS=vasoactive-inotropic score

**Table 2:** Major adverse events (MAE) observed within 48 hours after surgery.

MAE	N (%)
Cardiac arrest	19 (9.8%)
Death	13 (6.7%)
Reoperation, unplanned	8 (4.1%)
Low cardiac output syndrome	6 (3.1%)

Counts of MAE sum up to more than 31 because one patient may have had more than one MAE. Percentages refer to the whole sample of 194 patients.

Despite experiencing MAE, some patients also had minor/moderate adverse events and complications (92 events) — pleural effusion, cardiac arrhythmia, reintubation, pneumonia, and mechanical ventilation > 7 days were the most frequent events. They are described in Table 3.

**Table 3:** Postoperative complications, according to the occurrence of a major adverse event (MAE) within 48 hours after surgery.

Complication	Control (n=163)	MAE (n=31)	Total (n=194)
Pleural effusion	2 (1.2%)	22 (70.9%)	24 (12.4%)
Cardiac arrhythmia	5 (3.1%)	6 (19.3%)	11 (5.7%)
Reintubation	-	7 (22.6%)	7 (3.6%)
Pneumonia	2 (1.2%)	3 (9.7%)	5 (2.6%)
Mechanical ventilation, > 7 days	2 (1.2%)	3 (9.7%)	5 (2.6%)
Bleeding	3 (1.8%)	1 (3.2%)	4 (2.1%)
Seizure	1 (0.6%)	3 (9.7%)	4 (2.1%)
Unplanned extubation	-	3 (9.7%)	3 (1.6%)
Sepsis and septic shock	-	3 (9.7%)	3 (1.6%)
Pneumothorax	-	3 (9.7%)	3 (1.6%)
Chylothorax	-	2 (6.5%)	2 (1.0%)
Malignant hyperthermia	1 (0.6%)	-	1 (0.5%)
Infectious endocarditis	-	1 (3.2%)	1 (0.5%)
Pulmonary hypertension	-	1 (3.2%)	1 (0.5%)
Catheter-based procedure, unplanned	-	1 (3.2%)	1 (0.5%)

**Fig. 2:**
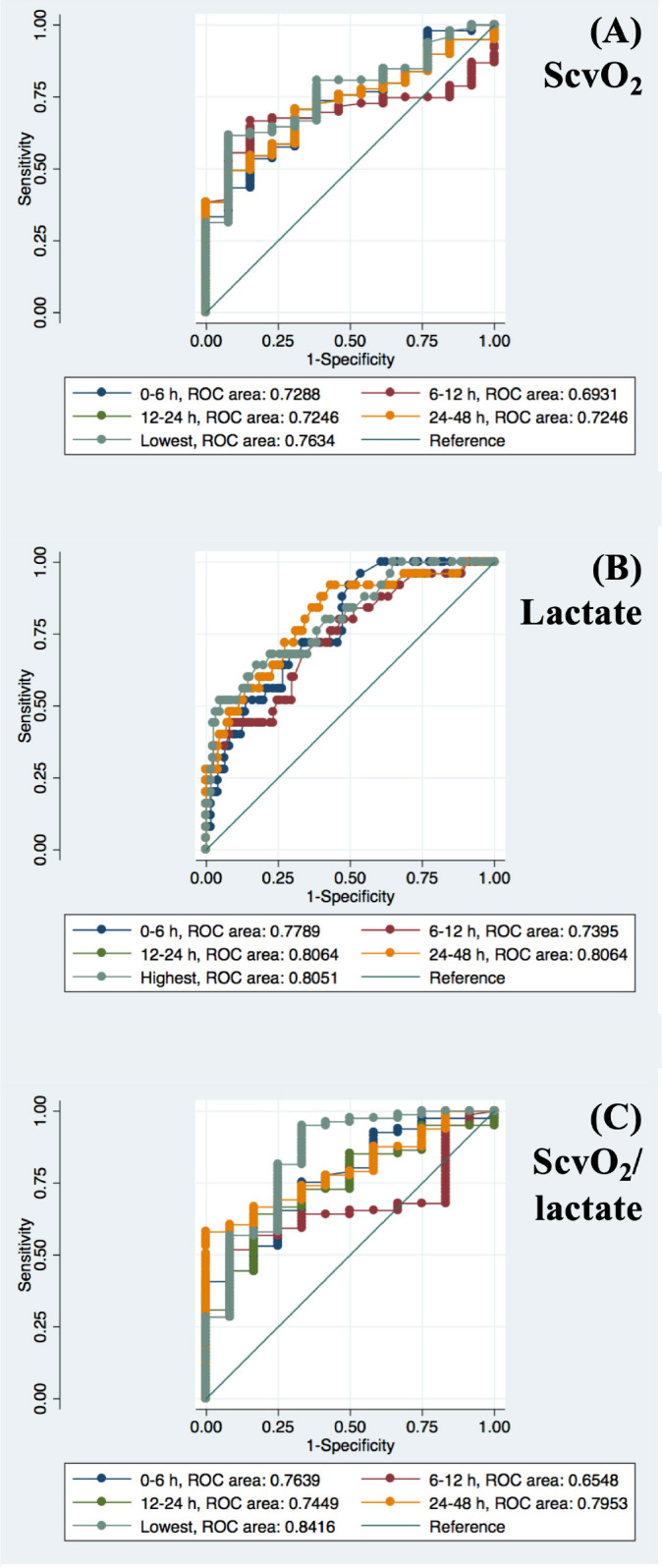
Receiver operating characteristics (ROC) curves with respective areas under the ROC curve of (A) central venous oxygen saturation (ScvO2), (B) arterial lactate, and (C) ScvO2/lactate ratio in children within the first 48 hours after congenital heart surgery for prediction of a major adverse event.

The mortality for patients who experienced a MAE was significantly higher than that of patients in the control group (45.1% *vs*. 6.7%, *P*<0.001). Patients who experienced MAE had significantly higher lactate levels and significantly lower ScvO_2_ and ScvO_2_/lactate ratio values than those not experiencing MAE at all time frames after surgery, except for lactate between six and 12 hours (Table 4). For ScvO_2_, the AUC in all time frames were good discriminants of MAE. The best AUC for ScvO_2_ was obtained with the lowest value between zero and 48 hours after surgery (0.763) (Figure 2A). For lactate, all AUC were good discriminants of MAE, and the best AUC was obtained with the values between 12 and 48 hours (0.806) (Figure 2B). For ScvO_2_/lactate ratio, values in all time frames were also good discriminants of MAE. The best AUC for ScvO_2_/lactate ratio was the lowest value between zero and 48 hours after surgery (0.841) (Figure 2C). ScvO_2_/lactate ratio had the higher AUC for prediction of MAE.

**Table 4:** Values of postoperative arterial lactate, central venous oxygen saturation (ScvO_2_), and ScvO_2_/lactate ratio, according to the occurrence of a major adverse event (MAE) within 48 hours after surgery.

	Marker	Control (n=163)	MAE (n=31)	*P*-value
ScvO_2_ (%)	0-6 h	59±23	48±25	0.031
	6-12 h	58±23	50±10	0.157
	12-24 h	59±21	47±18	0.023
	24-48 h	59±21	47±18	0.023
	Lowest value	54±23	40±25	0.005
Lactate (mmol/L)	0-6 h	2.8±2.2	7.7±6.7	< 0.001
	6-12 h	2.6±1.7	7.4±6.5	< 0.001
	12-24 h	2.2±1.4	5.7±5.4	< 0.001
	24-48 h	2.2±1.4	5.7±5.4	< 0.001
	Highest value	3.3±2.7	10.7±7.8	< 0.001
ScvO_2_/lactate ratio	0-6 h	29.9±18.6	14.0±10.6	< 0.001
	6-12 h	31.1±23.4	15.4±10.3	0.009
	12-24 h	37.1±22.0	15.3±11.9	< 0.001
	24-48 h	43.9±25.6	18.1±13.5	< 0.001
	Lowest value	24.8±18.2	8.8±9.5	< 0.001

Table 5 presents a comparison between different cutoff points for ScvO_2_/lactate ratio. All cutoff points rendered good NPV, but very low PPV and variable sensitivity and specificity. The lower cutoff (< 5) increases specificity at the cost of a low sensitivity, while the higher cutoff (< 17) increases sensitivity at the cost of low specificity. The intermediate cutoff point (< 9) yields very good NPV, and low sensitivity (61%) with fair specificity (85%). The cutoff < 5 had the best accuracy (87%). According to each cutoff, the relative risks of a patient with a ScvO_2_/lactate ratio < 5, < 9, or < 17 experience a MAE were 6.33 (95% confidence interval [CI] 3.60-11.2), 5.40 (95% CI 2.85-10.2), and 3.64 (95% CI 1.77-7.47), respectively. Based on these numbers, having a ScvO_2_/lactate ratio < 5 at any time within 48 hours after surgery is associated with a six-fold increased risk of MAE, with accuracy of 87% and specificity and NPV > 90%.

**Table 5:** Comparison of diagnostic values of different cutoff points for the lowest central venous oxygen saturation (ScvO_2_) to arterial lactate ratio for prediction of major adverse events.

Cutoff	Sensitivity (%)	Specificity (%)	PPV (%)	NPV (%)	Accuracy (%)
< 5	48	94	60	91	87
< 9	61	85	43	92	81
< 17	71	66	28	92	67

NPV=negative predictive value; PPV=positive predictive value

## DISCUSSION

We have shown that ScvO_2_/lactate ratio can accurately predict the occurrence of MAE within 48 hours after pediatric congenital heart surgery, with very good NPV, but poor PPV. Thus, our hypothesis was not confirmed. This means that ScvO_2_/lactate ratio > 5 is best suitable for identifying patients at low risk of MAE. We have also confirmed that ScvO_2_/lactate ratio is superior to either variable alone for this purpose.

The need for predicting tools for the occurrence of MAE is not new. Many studies have focused on lactate clearance, time of elevated lactate, cardiopulmonary bypass (CPB) duration, inflammatory markers, and other markers, and emphasis have been put in predicting LCOS, but an ideal predictive tool has not been found yet, as of today.

One of the first attempts was made in a study with 63 neonates undergoing heart surgery for transposition of the great arteries, in which high postoperative levels of cardiac troponin T, interleukin (IL)-6, and IL-8 were associated with LCOS^[6]^. In 46 children submitted to open heart surgery, our group showed that preoperative N-terminal pro-B-type natriuretic peptide plus postoperative IL-8 or platelet count were independent predictors of LCOS (sensitivity 93%, specificity 75%, PPV 87%, and NPV 86%), while CPB duration or postoperative IL-8 plus postoperative cardiac troponin I (cTnI) were independent predictors of death (sensitivity 100%, specificity 65%, PPV 38%, and NPV 100%)^[7]^. In 99 consecutive children undergoing open heart surgery for congenital heart disease, a cTnI level > 13 ng/mL four hours postoperatively predicted LCOS with sensitivity of 0.78, specificity of 0.72, and AUC of 0.75. In addition, cTnI was the only predictive variable associated with LCOS in multivariate logistic regression analysis^[8]^. In a study of 312 post CPB neonates, an oxygen delivery index had an AUC of 0.79, showing good prediction of low ScvO_2_, as a surrogate for LCOS^[9]^. In 117 children submitted to open heart surgery, the VIS score at two hours after CPB was an independent predictor of LCOS^[10]^. A LCOS score (tachycardia, oliguria, toe temperature < 30 °C, volume administration > 30 mL/kg/day, decreased near infrared spectroscopy measurements, hyperlactatemia, and need for vasoactive/inotropes in excess of milrinone at 0.5 µg/kg/min) showed an AUC for a composite morbidity (prolonged mechanical ventilation, new infection, cardiopulmonary arrest, neurologic event, renal dysfunction, necrotizing enterocolitis, and extracorporeal life support) of 0.83 in 55 patients undergoing pediatric open heart surgery^[11]^. Later, a modified LCOS score was superior to the inadequate oxygen delivery index in predicting adverse events linked to LCOS in 536 bypass pediatric operations for congenital heart defects^[12]^.

Only a few studies focused on the detection of MAE, a more comprehensive outcome than LCOS. Duke et al.^[2]^ studied a cohort of 90 children submitted to heart surgery and investigated the predictive value of several variables for MAE. They found an incidence of MAE of 13.3% (ours was 18.9%), and the independent predictors of MAE were lactate (measured at intensive care unit admission), CPB duration, lactate and CO_2_ difference (both measured four hours after admission), and lactate and base deficit (both measured eight hours after admission). They did not study ScvO_2_/lactate ratio specifically. Kalyanaraman et al.^[13]^ studied 129 children submitted to heart surgery and evaluated the predictive value of repeated measurements of arterial lactate for MAE. Moreover, they looked at the amount of time that lactate was > 2 mmol/L (“lactime”). They concluded that “lactime” had 98% sensitivity, but only 60% PPV for a postoperative MAE. Later, Schumacher et al.^[14]^ studied 231 infants undergoing cardiac surgery, and found that a lactate increase rate of 0.6 mmol/L/h had very good discriminatory ability (AUC 0.89) with a sensitivity of 90%, specificity of 84%, PPV of 34%, and NPV of 99% for a poor outcome (death, need for extracorporeal support, and dialysis). More recently, in a cohort of 257 pediatric patients undergoing cardiac surgery, the incidence of MAE was 19% and mortality was 13%. The predictors of MAE were cyanotic congenital heart disease, longer CPB duration (> 120 minutes), high inotropes (at least two, at high doses), and increase in lactate > 0.75 mmol/L/h or more in the first 24 hours (odds ratio 37.1, 95% CI 10.1-136.3)^[15]^.

Before our study, only one study assessed the predictive value of the ScvO_2_/lactate ratio for MAE. Seear et al.^[1]^ showed, in 52 children submitted to heart surgery, that a ScvO_2_/lactate ratio < 5 predicted MAE with PPV of 93.8%, sensitivity of 78.9% and specificity of 90.5% (NPV was not reported). They also showed that the PPV of ScvO_2_/lactate ratio was higher than those of a ScvO_2_ < 40% (58.3%) or a lactate > 8 mmol/L (63.6%) alone. In our study, using the same cutoff point (< 5), NPV was higher than PPV, specificity was high, but sensitivity was too low. These results suggest that calculating the ScvO_2_/lactate ratio when caring for a pediatric patient right after open heart surgery is an easy, quick, and inexpensive tool that may help physicians to identify patients at low risk of MAE, allocating more resources to those at higher risk.

### Limitations

The limitations of our study include: (a) retrospective data, implying that some MAE may have not been properly recorded and that ScvO_2_ and lactate were not consistently measured; and (b) the significant heterogeneity of heart conditions and surgeries, which compromises generalizability to other centers and populations. Therefore, our results need to be taken with caution.

## CONCLUSION

In conclusion, ScvO_2_/lactate ratio > 5 can accurately identify patients at low risk of MAE after pediatric congenital heart surgery, with very good specificity and NPV, but poor sensitivity and PPV.

**Table:** Authors' roles & responsibilities

VHSR	Substantial contributions to the design of the work; and the acquisition and analysis of data for the work; drafting the work; final approval of the version to be published
PHM	Substantial contributions to the conception of the work; revising the work critically for important intellectual content; final approval of the version to be published
FC	Substantial contributions to the conception or design of the work; and analysis of data for the work; drafting the work; final approval of the version to be published
